# Origin and Health Impacts of Emissions of Toxic By-Products and Fine Particles from Combustion and Thermal Treatment of Hazardous Wastes and Materials

**DOI:** 10.1289/ehp.8629

**Published:** 2006-01-26

**Authors:** Stephania A. Cormier, Slawo Lomnicki, Wayne Backes, Barry Dellinger

**Affiliations:** 1 Department of Biological Science and; 2 Department of Chemistry, Louisiana State University, Baton Rouge, Louisiana, USA; 3 Department of Pharmacology, Louisiana State University Health Sciences Center, Baton Rouge, Louisiana, USA

**Keywords:** cardiovascular health, environmental health, fine and ultrafine particulate matter, persistent free radicals, respiratory health, thermal remediation

## Abstract

High-temperature, controlled incineration and thermal treatment of contaminated soils, sediments, and wastes at Superfund sites are often preferred methods of remediation of contaminated sites under the Comprehensive Environmental Response, Compensation, and Liability Act of 1980 and related legislation. Although these methods may be executed safely, formation of toxic combustion or reaction by-products is still a cause of concern. Emissions of polycyclic aromatic hydrocarbons (PAHs); chlorinated hydrocarbons (CHCs), including polychlorinated dibenzo-*p*-dioxins and dibenzofurans; and toxic metals (e.g., chromium VI) have historically been the focus of combustion and health effects research. However, fine particulate matter (PM) and ultrafine PM, which have been documented to be related to cardiovascular disease, pulmonary disease, and cancer, have more recently become the focus of research. Fine PM and ultrafine PM are effective delivery agents for PAHs, CHCs, and toxic metals. In addition, it has recently been realized that brominated hydrocarbons (including brominated/chlorinated dioxins), redox-active metals, and redox-active persistent free radicals are also associated with PM emissions from combustion and thermal processes. In this article, we discuss the origin of each of these classes of pollutants, the nature of their association with combustion-generated PM, and the mechanisms of their known and potential health impacts.

In 1980, Congress passed the Comprehensive Environmental Response, Compensation, and Liability Act (1980; more commonly known as Superfund) that was designed to deal with abandoned and uncontrolled waste sites. Two classes of presumptive remedies exist to deal with Superfund sites: containment and treatment. Containment simply prevents the spread of contaminants to the soil, air, and water, whereas treatment employs technologies to rid the area of contaminants. The treatment regimens for soils and solid wastes most often employed are some form of combustion or thermal treatment, including *a*) incineration, which uses high temperatures to degrade contaminants; *b*) on-site thermal destruction, which is, in effect, a low-grade incineration process; and *c*) thermal desorption, in which toxic chemicals are first desorbed from the medium, collected, transported off-site, and then usually incinerated.

Combustion and thermal processes are dominant sources of air pollution. Although much attention is still paid to their contribution to priority air pollutants [i.e. ozone, volatile organic compounds (VOCs), and nitrogen oxides (NOx)], they also produce chronically toxic products of incomplete combustion (PICs). The greenhouse gas carbon dioxide is a product of complete combustion of carbon, and the ozone promoter NO_x_ is a product of complete combustion of nitrogen. However, chronically toxic organic pollutants, such as benzene, polychlorinated dibenzo-*p*-dioxins and dibenzofurans (PCDD/Fs), acrylonitrile, and methyl bromide, are products of incomplete combustion of carbon, carbon and chlorine, carbon and nitrogen, and carbon and bromine compounds, respectively. Although these toxic combustion by-products are formed in many types of combustion and thermal processes, they have historically been of particular concern for incineration of hazardous wastes and soils/sediments contaminated with hazardous wastes. For this reason, on-site incineration, defined as direct contact of the waste material with a flame, has come into disfavor. Instead, thermal destruction or desorption (in which the waste does not directly contact the flame) has been frequently substituted. Unfortunately, low- or moderate-temperature treatment has the potential to form more toxic by-products than does incineration.

Although some of these pollutants are emitted in the gas phase of combustion, they are frequently associated with fine and ultra-fine particulate matter (PM). Fine particles, PM_2.5_, are particles having an aerodynamic diameter of < 2.5 μm, and ultrafine particles, PM_0.1_, have an aerodynamic diameter of < 0.1 μm. Because so many pollutants are associated with fine PM and because fine PM has been strongly implicated in pulmonary and cardiovascular disease, much research has focused on its health impacts. Multiple theories have been proposed for the observed health impacts of fine and ultrafine PM; however, increasing evidence shows induction of oxidative stress as the progenitor of many of the observed illnesses. Polyclycic aromatic hydrocarbons (PAHs); chlorinated hydrocarbons (CHCs), including PCDD/Fs; brominated hydrocarbons (BHCs), including mixed brominated/chlorinated dioxins and furans (PXDD/Fs); toxic and redox-active metals; and persistent redox-active free radicals have been found to be associated with combustion-generated PM and have been suggested as responsible agents for one or more observed health impacts.

In this article, we discuss the origin and emissions of toxic combustion by-products and their potential health impacts when associated with combustion-generated fine and ultrafine PM.

## The Nature and Origin of Emissions of Toxic Combustion By-products

The nature of combustion by-products is determined by the chemicals that are treated and the conditions under which they react. Although incinerators, catalytic oxidizers, thermal desorbers, and accidental fire scenarios are quite different from an engineering perspective, the underlying reaction chemistries that form pollutants are closely related.

### Origin of emissions: a chemical reaction zone theory

**Figure 1:**
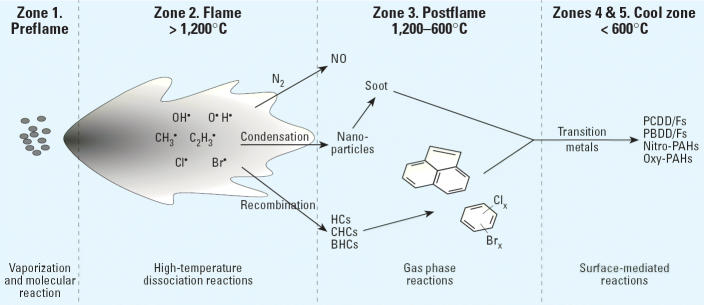
Combustor reaction zones. Zone 1, preflame, fuel zone; zone 2, high-temperature, flame zone; zone 3, postflame, thermal zone; zone 4, gas-quench, cool zone; zone 5, surface-catalysis, cool zone. PBDD/Fs, polybrominated dibenzo-*p*-dioxins and dibenzofurans. Reaction products from upstream zones pass through downstream zones and undergo chemical modifications, resulting in formation of new pollutants. Zone 2 controls formation of many “traditional” pollutants (e.g., carbon monoxide, sulfur oxides, and nitrogen oxides). Zones 3 and 4 control formation of gas-phase organic pollutants. Zone 5 is a major source of PCDD/Fs and is increasingly recognized as a source of other pollutants previously thought to originate in zones 1–4.

Toxic combustion by-products include two broad categories of organic pollutants that are defined under the Resource Conservation and Recovery Act of 1976. They are residual, undestroyed emissions of so-called principal organic hazardous constituents (POHCs) that are contained in the feed-stock and PICs that are formed during the thermal treatment. In addition, toxic metals may be vaporized and emitted; however, they more frequently react with oxygen or chlorine, resulting in a change in chemical form and oxidation state. Few, if any, organic components of the feed-stock survive direct contact with the flame, and other than soot, only minimal organic by-products are formed (Dellinger and Taylor 1998; Oppelt 1986). Thus, the vast majority of the observed pollutants in the effluent must be originating from chemistry occurring outside the flame. In fact, most of the pollutants are probably formed in the high-temperature, postflame zone or at even lower downstream temperatures as a result of surface-mediated reactions. In the most general sense, the mechanisms of pollutant formation and destruction are expected to be relatively consistent within a zone. This “zone model” allows for classification of the reactions occurring within a given zone (Dellinger and Taylor 1998) (Figure 1).

#### Zone 1, the preflame, fuel zone

This zone is characterized by a wide range of temperatures (from near ambient to 1,200°C), residence times on the order of 0.1 sec, and low excess air conditions. Because this zone occurs at the front end of the device, it creates new reaction intermediates by several low-energy, unimolecular reaction pathways such as hydrogen chloride elimination and carbon–halogen bond rupture that react further in the downstream zones.

#### Zone 2, the high-temperature, flame zone

This zone is characterized by temperatures of 1,000–1,800°C, at which essentially every organic compound will undergo complete conversion to its most thermodynamically stable end products, namely, carbon dioxide, water, hydrochloric acid, and nitric oxide. Under local pyrolysis conditions, soot is the dominant product. The flame zone generates large quantities of vaporized metals and chlorine that are very important reactants in subsequent zones. Observed organic pollutants are likely due to flow paths that pass through the periphery of the flame or flow eddies of poor fuel/air mixing (Cundy et al. 1989). These flow paths represent destruction “failure modes” of the flame and generate pockets that are more properly described as high-temperature thermal zones—that is, zone 3.

#### Zone 3, the postflame thermal zone

This is a chemistry-rich zone where various types of radical–molecule reactions occur. It is characterized by temperatures of approximately 600–1,100°C, residence times of a few seconds, and both oxygen-rich and oxygen-depleted regions. Experimental and modeling studies indicate that most pollutant formation in this zone occurs in oxygen-depleted pockets of poor waste–air mixing (Chang DPY et al., unpublished data; Cundy et al. 1989; Russell et al. 1989). Within this zone, most of the PAHs, higher-molecular-weight CHCs, brominated hydrocarbons (BHCs), and mixed bromo/chlorocarbons (XHCs) are formed by molecular growth pathways. This zone may also be where metals vaporized in the flame zone are condensed to ultrafine PM.

#### Zone 4, the gas-quench, cool zone

This zone exists downstream of the flame and post-flame zones and is characterized by either gradual or rapid quenching of the gas temperature. Residence times are long, > 10 sec, and oxygen concentrations vary from oxygen-depleted zones, due to combustion in upstream zones, to oxygen-rich zones if air in-leakage occurs. Partially oxidized products such as formaldehyde, chloroformaldehyde, and phosgene form by radical–oxygen association reactions (Russell et al. 1989). Nitrated products form via radical–molecule addition reactions involving NO_x_ generated in the flame zone (Schuetzle and Perez 1983). Hydrocarbons and chlorocarbons may also be partially oxidized in this zone, resulting in emissions including oxy-PAHs and oxychloro-PAHs (Rubey WA et al., unpublished data).

#### Zone 5, the surface-catalysis, cool zone

This zone is fundamentally different from the other four zones in that one must now consider the effects of surfaces at temperatures between 200 and 600°C. Reaction times for gas–surface reactions are a few seconds for entrained particulate or hours for deposited particles. PCDD/Fs have been shown to be formed in zone 5 (Altwicker et al. 1992; Gullett et al. 1994; Lomnicki and Dellinger 2003a, 2003b). However, many more pollutants potentially form as a result of surface catalysis via pathways including CHCs, BHCs, and XHCs; polybrominated dibenzo-*p*-dioxins and dibenzofurans (PBDD/Fs) and PXDD/Fs; partially oxidized hydrocarbons and CHCs (i.e., carbonyls, alcohols, organic acids, epoxides); and nitro-PAHs, oxy-PAHs, and oxychloro-PAHs (Addink et al. 1995; Lomnicki and Dellinger 2003a, 2003b; Wehrmeier et al. 1998). Most of the reactions necessary to form these products require a transition metal catalyst (Lomnicki and Dellinger 2003a, 2003b; Pieters et al. 1984). We now suspect that the zone 2 and 3 reactions that form nanoparticles of soot/fly ash also transform metals into catalytically active forms and catalyze the formation of new toxic by-products in zone 5. Once formed in zone 5, the pollutants are emitted into the atmosphere because temperatures are too low to result in their destruction.

Incinerators and accidental fires contain all these zones. Thermal destruction devices contain only zones 3, 4, and 5. Thermal desorbers consist of low-temperature components of zone 3 as well as zones 4 and 5. Catalytic oxidizers consist of zones 4 and 5 only. The omission of zone 2 in most ways increases the probability of pollutant emissions by allowing all of the waste to react in zones 3–5, rather than destroying a large portion of it in the flame zone. Unfortunately, nonincineration, thermal technologies, and fires are not subject to the same strict testing and regulatory scheme as are incinerators. Consequently, most emissions of toxic combustion by-products from these sources remain uncontrolled.

### The nature of emissions: precursors and thermal reactions

We must be concerned with not only the level of emissions but also their toxicity and bioavailability as determined by the form in which they are emitted. However, the problem is not intractable, for three reasons: *a*) Only a limited number of products form from the direct oxidation or pyrolysis of a given compound; *b*) in addition to by-products from specific precursors, full-scale emissions characterizations and pilot-scale and laboratory studies have shown that there are certain “ubiquitous” by-products that form regardless of the waste being burned; and *c*) the conditions under which pollutant-forming reactions occur are well defined within the zone theory of pollutant formation.

These principles suggest that characterization of toxic combustion by-products may be studied in a systematic scientific manner. In addition to PAHs that are formed in virtually every combustion source, we now believe that the principal classes of pollutants from the combustion/thermal degradation of hazardous wastes are *a*) fine and ultrafine PM, *b*) CHCs/BHCs, and *c*) persistent radicals.

#### Fine and ultrafine PM

Ultrafine PM, or nanoparticles, is formed largely by combustion sources as primary PM emissions or as secondary particles formed by atmospheric chemical reactions of combustion emissions of sulfur and nitrogen oxides (Donaldson et al. 1998). Nanoparticles are not efficiently captured by air pollution control devices, are transported over long distances, and penetrate deep into the respiratory system, all of which enhance the potential negative health impacts (D’Alesio et al. 1999; Kauppinen and Pakkanen 1990).

**Figure 2:**
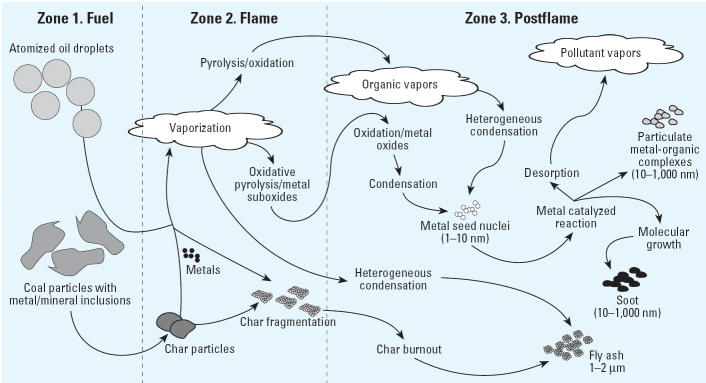
Nanoparticle formation/growth and mediation of pollutant-forming reactions in combustion systems. The combustor reaction zones described in Figure 1 effect particle formation as well as gas-phase pollutant formation. Metals and other refractory compounds are vaporized in the flame zone. They can recondense as cluster or seed nuclei in the postflame zone, where they catalyze further particle growth and pollutant formation in the cool zones.

Metals are vaporized in the flame zone and subsequently nucleate to form small metal nanoparticles or condense on the surfaces of other nanoparticles in transit to the postflame (thermal reaction) zone (Figures 1, 2). Under pyrolytic or oxidative pyrolysis conditions at temperatures above approximately 600°C (zone 2), the metal seed nuclei promote reactions with gas-phase organic species to form a carbonaceous layer, resulting in nanoparticle growth (zones 2 and 3). Below approximately 600°C, under primarily oxidative and oxidative pyrolysis (thermal reactions in the presence of trace quantities of oxygen) conditions, the metal nuclei or surface-condensed metals initiate formation of new gas-phase and PM-associated pollutants (zones 3–5).

Elemental carbon (mostly soot) and organic carbon (the myriad of organic chemicals) account for more than half of these particles. Although approximately 80% of the organic carbon is extractable, only 12% is chemically resolved (Rogge et al. 1993). PAHs, oxy-PAHs, alkanes, organic acids, and macromolecular species similar to humic acid make up most of the identified chemicals. These airborne particles also contain percent (e.g., iron, potassium, silicon) and part-per-million (e.g., copper, nickel, zinc) concentrations of transition, alkali, and other toxic metals. Redox-active metals (e.g., iron and copper) and organics (e.g., PAHs, oxy-PAHs, and semiquinones) have been implicated in the biological activity of airborne fine and ultrafine PM (Carter et al. 1997; Costa and Dreher 1997; Kennedy et al. 1998; Smith et al. 2000). Unfortunately, the organic fraction remains largely uncharacterized, and there are few to no data on speciation of metals and the presence of metal–organic complexes that undoubtedly exist in these particles.

#### Emissions of CHCs and BHCs

The combustion and thermal reactions of CHCs are of particular interest because *a*) they constitute most of the toxic components of hazardous wastes, *b*) they are often quite refractory, and *c*) they form other highly toxic aliphatic and olefinic CHCs, chlorinated PAHs, and PCDD/Fs.

Trichloroethylene produces a wide range of by-products, including hexachlorobenzene, chlorinated PAHs, and the perchlorinated analogue of the highly carcinogenic butadiene (Avakian et al. 2002). CHCs also form as byproducts in zone 5 by surface-mediated reactions. This finding, along with the resistance of CHCs to oxidation, suggests that the formation of PAHs and chlorinated PAHs (ClPAHs) may be more facile in halocarbon combustion systems than in hydrocarbon systems.

Numerous research studies have definitively demonstrated that PCDD/Fs are formed in almost any combustion or thermal device if there are sources of carbon and chlorine along with a transition metal to catalyze chlorination and condensation reactions (Dellinger and Taylor 1998; Froese and Hutzinger O 1996). Three general pathways of formation have been proposed: *a*) *de novo* formation (200–500°C), in which carbon in soot or fly ash acts as reagent to form PCDD/Fs by chlorination/ oxidation of “dioxin-like” structures that inherently exist in a carbon matrix (Altwicker 1996; Hell et al. 1997; Huang and Buekens 1996; Stieglitz 1998); *b*) transition-metal, surface-catalyzed formation (200–500°C) from PCDD/F precursors such as chlorinated phenols and chlorinated benzenes (Altwicker 1996; Froese and Hutzinger O 1996; Ghorishi and Altwicker 1996; Lomnicki and Dellinger 2003a, 2003b); and *c*) gas-phase, radical–molecule reactions ( > 600°C) of chlorinated phenols, chlorinated benzenes, and polychlorinated biphenyls (Lenoir et al. 1998; Louw and Ahonkhai 2002). Field studies suggest that gas-phase pathways are responsible for about 30% of total PCDD/F emissions, with the remainder due to surface-mediated pathways. Any source containing a hydrocarbon, a transition metal, and a source of chlorine (organic or inorganic) will form PCDD/Fs if the temperature is ever raised above 200°C (Altwicker 1996; Froese and Hutzinger M 1996; Ghorishi and Altwicker 1996; Lomnicki and Dellinger 2003a, 2003b).

There is a growing recognition that BHCs, including PBDD/Fs, are formed and emitted during the thermal treatment of brominated flame retardants in fabrics and plastics and electronic materials (E-materials and E-wastes), frequent contaminants at Superfund sites.

Until recently, BHCs have received little attention primarily because of difficulty of analysis, lack of available analytical standards, and a paucity of health effects data. However, recent findings suggest that brominated flame retardants as well as PBDD/Fs are highly toxic (Lenoir et al. 2001). Computer motherboards contain an incredible amount (~50% per unit) of bromine (Sakai et al. 2001). Analysis of the effluent from an E-waste incinerator reveals that the effluent contains 4.6–7.6 mg of copper per dry standard cubic meter (dscm). This is significant because it is well established that copper catalyzes the formation of PCDD/Fs from CHCs in combustion systems, and the same catalytic behavior is expected from BHCs. Recent experimental studies have shown that BHCs, PBDD/Fs, XHCs, and PXDD/Fs are formed from combustion of E-materials (Lemieux and Stewart 2004). It is clear that our understanding of the environmental hazards of emissions of XHCs is only in its infancy, and further progress is hindered by lack of understanding of the basic combustion chemistry and availability of analytical standards for toxicologic, chemical, and combustion evaluation.

#### Emissions of persistent free radicals

Reports of persistent radicals in coals, chars, and soots date back to the 1950s (Ingram et al. 1954; Lyons and Spence 1960; Lyons et al. 1958; Uebersfeld et al. 1954). Although a link between free radicals in these samples and health impacts was suspected, their potential health impacts were not recognized because they were thought to be “inaccessible to cells and too stable to play any part in car-cinogenesis” until the publication of a series of papers by Pryor and colleagues demonstrating the viability of catalytic cycles involving semi-quinone radicals (Pryor and Squadrito 1995; Pryor et al. 1976; Squadrito et al. 2001).

However, we have recently found that persistent free radicals are present in combustion-generated fine and ultrafine PM and that these radicals induce DNA damage (Dellinger et al. 2001). Using electron paramagnetic resonance, we have found that combustion of 10 different fuels and CHCs produced semiquinone-type radicals that are stabilized on the particle surfaces (Dellinger et al. 2001; Squadrito et al. 2001). Semiquinone radicals are known to undergo redox cycling to produce biologically damaging superoxide and hydroxyl radicals.

Because the principal source of airborne fine PM is combustion, and these sources generate free radicals, we examined samples of PM_2.5_ from six cities and found large quantities of radicals with characteristics similar to semiquinones (Dellinger et al. 2001). Aqueous extracts of combustion-generated PM and PM_2.5_ samples induced damage to DNA in human lung epithelial cells and myeloid leukemia cells. PM_2.5_-mediated DNA damage was abolished by superoxide dismutase, catalase, and desferoxamine, implicating the superoxide radical, hydrogen peroxide, and the hydroxyl radical in the reactions inducing DNA damage. Identical DNA damage was caused by incinerator bottom ash (Dellinger 2003).

We believe that the source of this damage is a surface-associated semiquinone-type radical. Semiquinones are relatively nonreactive with O_2_ due to resonance stabilization (Berho and Lesclaux 1997; Wiater-Protas and Louw 2001). When a semiquinone is adsorbed on a surface, additional stability may be imparted to the radical if the adsorption site is an electron acceptor (Kodomari et al. 1988). The presence of semiquinone-type radicals on combustion-generated PM is significant and suggests a previously unrecognized origin of the health effects attributed to fine PM.

## Health Effects of Toxic Combustion By-products

### Routes of exposure and distribution: size matters

Combustion of hazardous wastes results in pollution that exists in a gaseous, liquid, and/or solid particle state suspended in air. A crude characterization of suspended pollutants uses the mean diameter of the suspended particles and varies from a few nanometers to several micrometers. The coarse fraction of suspended airborne pollutants originates from windblown dust, crushing and grinding operations, materials handling, and/or atmospheric abrasion of even larger particles. The aerodynamic diameter of inhalable coarse PM ranges from 2.5 to 10 μm (i.e.,PM_10_). Combustion, on the other hand, typically generates smaller PM < 2.5 μm in diameter (i.e., PM_2.5_). Finally, ultrafine PM, or nanoparticles, form both in combustion sources and in atmospheric processes through condensation and molecular growth pathways and are < 100 nm (i.e., PM_0.1_) in diameter.

**Figure 3:**
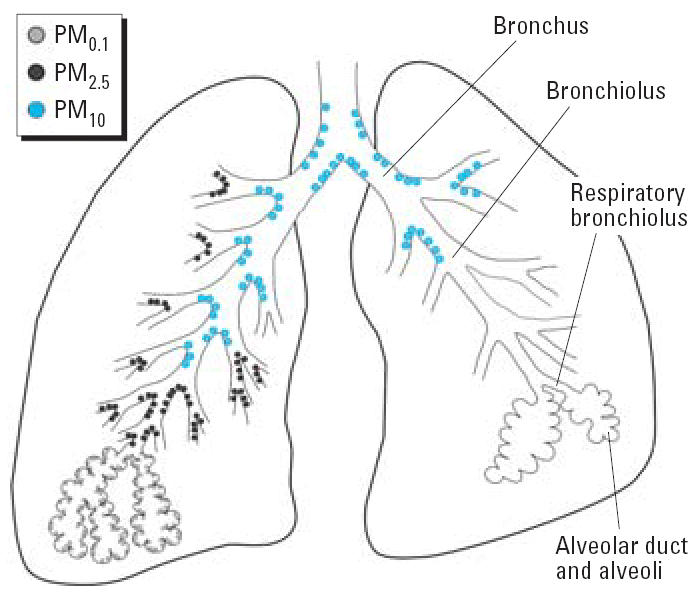
Distribution of PM in the airways. PM ≥10 μm in diameter enter the nose and mouth. The thoracic fraction, PM_10_, passes the larynx and penetrates the trachea and bronchial regions of the lung, distributing mainly at pulmonary bifurcations.The respirable fraction, PM_2.5_, and ultrafine PM, PM_0.1_, enter the nonciliated alveolar regions and deposit deep within the lungs.

Particles are deposited in the respiratory tract, and deposition is directly proportional to aerodynamic diameter of the particles (Figure 3). PM_10_ deposits mainly in the upper respiratory tract and may be cleared by mucociliary actions. PM_2.5_ and PM_0.1_ penetrate the alveolar regions of the lung, where the ultrafine PM rapidly penetrates the epithelium (Oberdorster 2001). Clearance of fine and ultrafine PM is mediated mainly by phagocytic activity and particle dissolution (Wagner and Foster 1996).

The ability of PM_0.1_ to translocate to the pulmonary interstitium suggests that these particles have a significant impact on the health of other organ systems (Nemmar et al. 2001). Indeed, studies using radiolabeled 2,3,7,8-tetrachlorodibenzo-*p* –dioxin ([^3^H]TCDD) have clearly demonstrated that inhalation, ingestion, or dermal absorption results in major tissue deposits of [^3^H]TCDD in the liver and fat (Diliberto et al. 1996) and suggest that multiple routes of exposure occur and that these exposures lead to multiple organ and systemic effects.

Ambient air pollution is a complex mixture of volatiles and particulates arising from various sources, including vehicular exhaust, flaring of hydrocarbons at refineries, coal burning at power plants, and thermal treatment of hazardous wastes at Superfund sites. A larger number of epidemiologic studies have documented associations between air pollution, specifically PM_2.5_ and PM_0.1_, and acute health effects (Burnett et al. 2000; Ostro et al. 1996; Peters et al. 2001; Pope et al. 1999). However, very little is known about the health effects associated with exposure to the by-products produced from the combustion of hazardous wastes. Thus, the following discussion is primarily based on a review of recent literature addressing the effects of air pollution on health effects.

### Pulmonary effects

#### Decreased lung function

Increases in ambient air pollution result in increased hospital admissions for numerous respiratory end points, including decreased lung function [i.e., reductions in peak flow and declines in forced expiratory volume in 1 sec (FEV_1_)], cough, and exacerbations of pulmonary disease states such as asthma and chronic obstructive pulmonary disease (Boezen et al. 1998; Pope 2000; Schwartz 1994; Timonen and Pekkanen 1997; Vedal et al. 1998). Interestingly, stratifying the results of some of these studies for sex demonstrated an increase in asthma attacks in girls compared with boys (Brunekreef et al. 1997; Oosterlee et al. 1996; Van Vliet 1997). However, none of these reports even postulates as to why females may be more susceptible to air pollution than are males.

#### Inflammatory responses

Exposure to airborne PM has been shown to elicit an acute inflammatory response (i.e., an influx of neutrophils and other inflammatory cells in the airway lumen and release of proinflammatory cytokines) in the lung (Carter et al. 1997; Fujii et al. 2001; van Eeden 2002). Effects of air pollution on pulmonary function are observed in various animal models, including rats, mice, and dogs (Henderson et al. 1988; Hiura et al. 1999; Nel et al. 2001; Saldiva et al. 1992). In a recent study, normal rats exposed to concentrated ambient air particles (PM_2.5_) for 3 consecutive days demonstrated a dose-dependent increase in pulmonary inflammation, as measured by increased neutrophil numbers in the bronchoalveolar lavage fluid (Saldiva et al. 2002). These data were supported by histopathology demonstrating an acute inflammatory response characterized by an influx of neutrophils into the central areas of the pulmonary acinus, hyperplasia of the alveolar epithelium, and macrophage accumulation in the alveolar spaces.

#### Immune responses

Data further suggest that ambient air pollution has the ability to modulate immune responses due to certain respiratory viral infections. PM_10_ exposure has been shown to interfere with the replication of respiratory syncytial virus (Kaan and Hegele 2003) and to lead to a decreased production of proinflammatory cytokines (Vincent et al. 1997), whereas exposure of rhinovirus-infected epithelial cells to moderate levels of air pollutants led to enhanced generation and release of proinflammatory cytokines (Spannhake et al. 2002). Although conflicting, these data suggest that air pollution modulates pulmonary inflammation due to certain viral infections *in vivo* and may be important in the exacerbation of respiratory inflammatory disease states such as asthma and chronic obstructive pulmonary disease.

Numerous epidemiologic studies have demonstrated increased mortality associated with increased levels of PM. On high-pollution days, the numbers of deaths due to respiratory viral infections such as pneumonia were disproportionately high (Schwartz 1994). In fact, hospitalization admissions for preschool-age children and elderly individuals were elevated almost 2-fold in communities where PM_10_ levels were above the 24-hr and annual National Ambient Air Quality Standards of 65 and 150 μg/m^3^ (Dockery and Pope 1996).

Several studies support these findings. In one such study, human alveolar macrophages (AMs) were isolated and subsequently exposed to PM. The AMs showed significant decreases in a number of receptors important for host defense such as CD11b and CD11c (important for phagocytosis of opsonized pathogens) and CD29 (important in neutrophil recruitment). Within 3 hr of exposure, the ability of AMs to generate reactive oxygen species (ROS; important in the killing of microorganisms) was markedly reduced, and within 18 hr, significant declines were observed in the phagocytic ability of AMs (Becker and Soukup 1998). More recent studies confirm that exposure to airborne particles from combustion of residual oil [residual oil fly ash (ROFA)] may alter AM function. For example, ROFA instilled into the trachea of rats before infecting them with *Listeria monocyto-genes* results in an increase in the phagocytic ability of AMs, decreased bacterial killing, and increased mortality (Antonini et al. 2002). These results correlated with a significant decrease in the production of nitric oxide by AMs. The demonstrated suppression of host defense mechanisms against *L. monocytogenes* is not specific to ROFA or PM, but has also been observed on exposure to sulfur-related air pollution, leading to long-term respiratory effects and to changes in AM-mediated particle clearance mechanisms (Kreyling et al. 1999).

Although the above studies agree with numerous other studies on ROFA and bacterial infectivity (Antonini et al. 2002; Hatch et al. 1985), they disagree with assessments of infectivity using other PM samples (Antonini et al. 2000; Yang et al. 2002). For example, AM function (i.e., phagocytosis and production of ROS) was actually enhanced in the lungs of animals exposed to crystalline silica and subsequently infected with *L. monocyto-genes* (Antonini et al. 2000). The reasons for this controversy are unclear; however, it is anticipated that the various components associated with the source of the PM are important in the observed effects.

Cumulatively, these data suggest that air pollution acts as an immunosuppressor, deflating the normal host response to pathogens and, in particular, the pulmonary immune response. Whether this is a result of decreased AM cell numbers, decreased AM phagocytic abilities, and/or diminished T-cell responses appears to depend on the chemical composition of the exposure.

#### Diminished lung function growth

Although effects on pulmonary function are obvious, long-term effects such as lung function growth in children are just being realized. Gauderman et al. (2002) followed a cohort of 1,678 fourth-grade schoolchildren from 12 different southern California communities over a period of 4 years. Each spring, a team of Children’s Health Study technicians obtained seven maximal forced expiratory maneuvers from each child as a measurement of pulmonary function. Air pollution in the 12 communities was monitored for the entire study period. Air-monitoring stations recorded hourly concentrations of ozone, PM_10_, and nitrogen dioxide levels. PM_2.5_ levels were obtained from 2-week filter samples. Investigators observed a negative correlation between pollution levels and pulmonary function for all pollutants examined. A significant negative correlation was observed between FEV_1_ growth rate and acid vapor (*p* = 0.03). Significant negative correlations between FEV_25–75%_ (the middle 25–75% of the FEV maneuver) were observed for acid vapor, nitrogen dioxide, PM_2.5_, and elemental carbon. Despite the large number of publications in this area, no resounding theory as to how ambient PM induces pulmonary dysfunction has surfaced.

### Cardiovascular effects

#### Increased cardiovascular events

Epidemiologic studies have also shown an increase in cardiovascular morbidity and mortality that is associated with increases in PM. In fact, cardiovascular death rates were higher than pulmonary death rates during peak episodes of air pollution (Pope et al. 1999). Numerous studies conducted within the United States and other countries, including Canada and Chile, have reported statistically significant, positive correlations between daily human cardiovascular events and exposure to fine PM in the atmosphere (Burnett et al. 1995; Dockery et al. 1993; Ostro et al. 1996).

Unfortunately, the epidemiologic data do not provide a clear description of the types of cardiac events observed. In fact, cardiovascular deaths in most of these studies were lumped into a single group, coronary heart disease (CHD), which was associated with increases in ambient PM concentration (Poloniecki et al. 1997; Schwartz and Morris 1995). However, CHD results from myocardial ischemia, arrhythmias, arthrosclerosis, thrombosis, and/or vascular spasm. This represents a major problem in determining the underlying cause of cardiovascular mortality associated with increased PM levels. The temporal association between cardiovascular hospitalizations/mortality and ambient PM seems to be relatively short (0–3 days), suggesting that increased cardiovascular morbidity/mortality is due to myocardial ischemia (Pekkanen et al. 2002), myocardial infarcts (Peters et al. 2001), and/or ventricular arrhythmias (Peters et al. 2000), and heart rate variability (Gold et al. 2000; Pope et al. 1999). Short-term exposures (< 2 hr) have been shown to increase the occurrence of myocardial infarction in people at risk of developing CHD (Peters et al. 2001). Numerous animal studies have been able to replicate most of the observed human responses to PM. These studies demonstrate that acute exposure to environmentally relevant PM induces cardiovascular effects, including changes in heart rates (Gordon et al. 1998; Pope et al. 1999); arrhythmias (Hoek et al. 2001); electrocardiographic abnormalities (Bloch et al. 1972); cardiomyopathic changes, including inflammatory infiltrates, fibrosis, and cardiac myocyte degeneration (Kodavanti et al. 2003); and progression of atherosclerotic lesions (Suwa et al. 2002).

#### Chronic cardiovascular inflammation

Long-term exposure studies (10 mg/m^3^ at 6 hr/day and 1 day/week for 16 weeks) in Wistar Kyoto rats demonstrated that PM induces both time- and dose-dependent myocardial injury (Kodavanti et al. 2003). Histopathology of the cardiac tissue revealed randomly distributed foci of inflammatory responses composed of mixed populations of neutrophils, lymphocytes, and macrophages and suggests a state of chronic active inflammation in the heart due to PM exposure. The myocardial injury was characterized by cardiac myocytes in various stages of degeneration. The degenerating cardiac tissue was associated with fibrosis and collagen accumulation of the interventricular septum and throughout the ventricles. Interestingly, examination of the pulmonary tissue showed a dose-and time-dependent accumulation of particle-laden AMs with no associated peribronchial or perivascular inflammation or pulmonary fibro-sis, suggesting that PM directly affects cardiovascular tissue. A recent study using dogs residing in polluted urban areas of southwestern Mexico City demonstrated numerous myocardial changes, including apoptotic myocytes and inflammatory infiltrates in the left and right ventricles and interventricular septum (Calderon-Garciduenas et al. 2001). Vascular changes were also noted in the dogs, including smooth muscle cell hyperplasia, deposition of PM in the media and adventia, and microthrombi in the capillaries and small arteries and veins.

Very little is known about how PM increases the risk of cardiovascular events. One hypothesis is that inhaled PM produces an acute cardiovascular event indirectly through the induction and perpetuation of inflammatory responses in the lung. The chemokines and cytokines released during this inflammatory response travel through the blood to the myocardium, where they are known to cause myocardial dysfunction, including myocardial infarction, atherosclerosis, and decreased contractility (Abe et al. 1993; DeMeules et al. 1992; Mann and Young 1994). Indeed, a systemic inflammatory response induced by PM has been demonstrated (van Eeden 2002). This systemic response elicited cytokine release from the lung into circulation and proliferative responses of bone marrow polymorphonuclear leukocytes. In conjunction with the systemic inflammation, it was noted that a progression of atherosclerotic plaques occurred on exposure to PM in animals susceptible to atherosclerosis.

An alternative hypothesis is that the inhaled PM is absorbed by the blood and translocated from the lung to the heart. Provocative data from a few investigators have begun to demonstrate the ability of PM_0.1_ to penetrate deeply into the lower respiratory tract, where it is capable of producing significant systemic effects (Salvi et al. 1999), and to diffuse from the lungs into the systemic circulation (Nemmar et al. 2001). Evidence for transport of PM from the lungs into circulation was noted, although not discussed, in the canine study, which demonstrated deposition of PM in the arteriolar blood vessels (Calderon-Garciduenas et al. 2001). PM transported via the vasculature, directly or indirectly, influences the cardiac myocytes, cardiovascular functioning, and/or hemodynamics through thrombus formation or changes in rhythm.

### Genotoxicity

Genotoxicity results in DNA mutations that affect *a*) only the individual’s DNA (i.e., somatic mutations), *b*) only the DNA of the individual’s progeny (i.e., germline mutations), or *c*) the DNA of both the individual and its progeny. Genotoxic events are often considered the most detrimental; however, cytotoxic events also result in changes to the physiologic functioning of the organ/cell, a predisposition to develop disease, and/or cell death and organ damage.

PM_2.5_ and combustion-generated PM contain exogenous free radicals that have been shown to induce DNA damage (Dellinger et al. 2001) and mutagens (Demarini et al. 1991; Houk et al. 1990; Watts et al. 1992). In one study, mice chronically exposed to the ambient air pollution of downtown São Paulo for 90 days showed a significant increase in the frequency of micronuclei (an indicator of DNA damage), which was associated with increased levels of carbon monoxide, nitrogen dioxide, and PM_10_ (Soares et al. 2003). Similar data were observed on exposure of human bronchial epithelial cells to 1,3-butadiene soot (Catallo et al. 2001). Also, both cytotoxic and genotoxic mutations may lead to cancer (Vineis and Husgafvel-Pursiainen 2005). Increased mutagenicity associated with combustion PM emissions appeared to depend on the incompleteness of combustion and reduced efficiency of pollution control equipment.

Investigations conducted in Hamilton Harbor, Ontario, Canada (an industrial area with two steel mills), suggest that PM from air pollution and combustion emissions is the principal factor responsible for eliciting genetic mutations (Somers et al. 2004). In particular, offspring of mice exposed to industrial combustion from Hamilton Harbor demonstrated an increased incidence (i.e., 1.5- to 2-fold higher expanded simple tandem repeat mutation rates than animals exposed to ambient air) of DNA mutation rates that were paternally derived. Intriguingly, these data are the first to implicate PM in the induction of mutations heritable by the subsequent generations (Somers et al. 2004) and imply that inhaled PM or their metabolized products are transported to germ cells (Samet and Pope 2003).

### Reproductive effects

Exposure to environmental pollutants has also been linked to adverse reproductive health. Some of the effects observed include developmental changes in the male reproductive tract, including testicular abnormalities, whereas other effects include reduced fecundity (i.e., reduced sperm quality and count, levels of testosterone, and embryo implantation) (Carlsen et al. 1992; Dallinga et al. 2002; Pflieger-Bruss and Schill 2000; Swan et al. 2000). Studies using organochlorines, which are found in the diet of Inuit tribes from the Arctic (Dewailly et al. 1993), have demonstrated decreased motility and diminished viability of sperm within 2 hr of exposure. If exposure occurred during *in vitro* fertilization, the investigators observed diminished sperm penetration of the oocyte and slower development to blastocyst rates (Campagna et al. 2002).

Likewise, decreases in female fertility have been observed on exposure to environmental air pollution (Mohallem et al. 2005). Female mice exposed to ambient air for 4 months displayed higher incidences of implantation failure and decreases in live-born pups. These differences in fertility were significant if exposures to ambient air pollution began at an early age (i.e., 10 days after birth). Cumulatively, these studies suggest that pollutants affect implantation and reduce fertility by damaging the germline cells.

### Intrinsic properties of the host

The health impact due to various environmental exposures is highly variable and depends on multiple parameters both intrinsic and extrinsic to the individual. For example, season and climate have been shown to have a potential role in the health impacts associated with ozone (Guo et al. 1999; Lee et al. 2003). It is also plausible that certain populations are more susceptible to adverse health effects on exposure such as the elderly, the developing fetus, or those with pre-existing disease states. Consequently, several investigators are focusing on the impact of exposure in groups of specific ages or with specific preexisting diseases (Zanobetti and Schwartz 2002; Zanobetti et al. 2000).

It is also clear that genotypic polymorphisms exist among individuals within populations and that genetic background is an important susceptibility factor for adverse health effects on exposure to emissions. Some of the genes implicated in adverse health effects on exposure to ozone and sulfate-associated PM are toll-like receptor 4 (Kleeberger et al. 2000), proinflamatory cytokines (Ohtsuka et al. 2000), and tumor necrosis factor-α (Yang et al. 2005). Linkage analysis data are strongly supported by experimental data demonstrating a role for these candidate genes in ozone and PM susceptibility (Cho et al. 2005; Kleeberger et al. 2000). In particular, a recent study demonstrated that variability in genes encoding enzymes that are members of the xenobiotic defense pathways determines lung cancer risk from indoor coal combustion emissions (Lan et al. 2000). Null genotypes for glutathione *S*-transferase M1 were associated with increased risk of lung cancer (2.3-fold increase).

## Outlook

Understanding the relationships between the origins, mechanisms of formation, nature of emissions, biological availability, and biological activity of toxic combustion by-products will require well-coordinated interdisciplinary research by biomedical, biological, chemical, and engineering researchers. Furthermore, establishing the nature of this link will require each group of researchers to go beyond their traditionally narrow veins of research and to integrate their understanding into a new field of research that could be referred to as health effects engineering science.

Inhalation of airborne fine and ultrafine PM has been identified as a major route of exposure to toxic combustion by-products; research should address this poorly understood area. From a combustion and environmental chemistry perspective, key research issues include the following:

- How are combustion-generated fine PM and ultrafine PM formed?
- How do their chemical properties differ from larger PM?
- What is the nature of association of chemicals with these particles?
- How is the chemical and biological reactivity of these chemicals changed by association with the particles?
- What is the role of PM-associated persistent free radicals in the environmental impacts of fine and ultrafine PM?

From a health effects perspective, key research issues associated with combustion-generated fine and ultrafine PM include the following:

- What is the role of PM on cell/organ functioning at initial sites of exposure?
- What is the bioavailability of these particles to other tissues?
- How are these particles translocated to these secondary sites, and do their chemical properties change en route?
- How does acute/chronic exposure lead to adverse organ pathophysiology? Is developmental timing of exposure important?
- What effect does exposure have on predisposing to disease states or on disease progression?
- Most important, what are the specific cellular and molecular mechanisms associated with airborne exposures?
